# Supplementary material for: Cryptococcus neoformans adapts to the host environment through TOR-mediated remodeling of phospholipid asymmetry
Source: Nat Commun. 2023 Oct 18;14:6587. doi: 10.1038/s41467-023-42318-y (PMC10584969; doi:10.1038/s41467-023-42318-y)
Supplement: Supplementary file 1 — Supplementary Information [file 41467_2023_42318_MOESM1_ESM.pdf]

Supplementary Information

***Cryptococcus neoformans* adapts to the host environment through TOR-mediated remodeling of phospholipid asymmetry**

Laura C. Ristow[1], Andrew J. Jezewski[1], Benjamin J. Chadwick[2], Mark A. Stamnes[3], Xiaorong Lin[2,4], and Damian J. Krysan[1,3,5]

[1]Department of Pediatrics, Carver College of Medicine, University of Iowa, Iowa City IA 52242; [2]Department of Plant Biology, University of Georgia, Athens, GA 30602; [3]Department of Molecular Physiology and Biophysics, Caver College of Medicine, University of Iowa, Iowa City IA 52242; [4]Department of Microbiology, University of Georgia, Athens, GA, 30602; and of [5]Microbiology and Immunology, Carver College of Medicine, University of Iowa, Iowa City IA 52242.

Corresponding author:

Damian Krysan (damian-krysan@uiowa.edu)

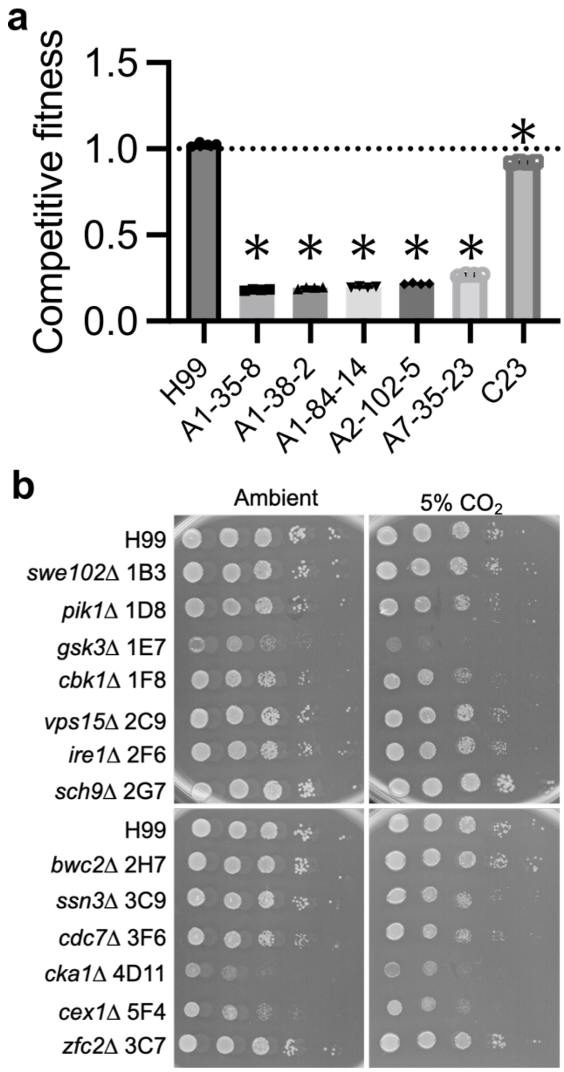

Supplementary Fig. 1

Competition assay sensitively detects phenotypes of CO_2-sensitive strains

a) Overnight cultures of mNeon-Green labeled H99 and CO_2-tolerant or CO_2-sensitive strains were combined in a 1:1 ratio and incubated at 30°C for 24 hours in ambient air or with 5% CO_2. Cell populations were characterized by flow cytometry and the percentage of mNeonGreen negative cells in 5% CO_2 normalized to those in ambient conditions to determine a competitive fitness score for each mutant strain as indicated. Bars represent the average and SEM of four biological replicates. An ordinary one-way ANOVA with Tukey's multiple comparisons test was performed using GraphPad Prism. Significance relative to H99 is represented. *, $P < 0.05$; $P_{A1-35-8}$=7.137e-13; P_{A138-2}=7.137e-13; $P_{A184-14}$=7.137e-13; $P_{A2-102-5}$=7.137e-13; $P_{A7-35-23}$=7.137e-13; P_{C23}=7.148e-13. b) Ten-fold serial dilutions from overnight cultures of indicated strains (plate location from kinase or transcription factor deletion library indicated) were spotted on solid RPMI medium with 165 mM MOPS, pH 7 at 30°C in ambient air or at 5% CO_2 for 48 hours. Plates are representative of two biological replicates. Source data are provided as a Source Data file.

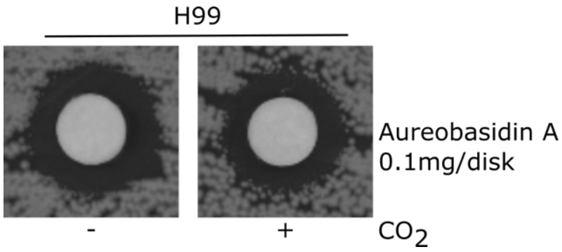

Supplementary Fig. 2

CO_2 exposure does not affect sensitivity to the IPC targeting antifungal aureobasidin

Cells from overnight cultures of H99 were spread on RPMI 1640 medium with 165 mM MOPS, pH 7. Sterile disks were placed on plates and 100 µg aureobasidin was added to each disk. Cells were incubated at 30°C for 48 hours in ambient air or 5% CO_2. Images are representative of two biological replicates.

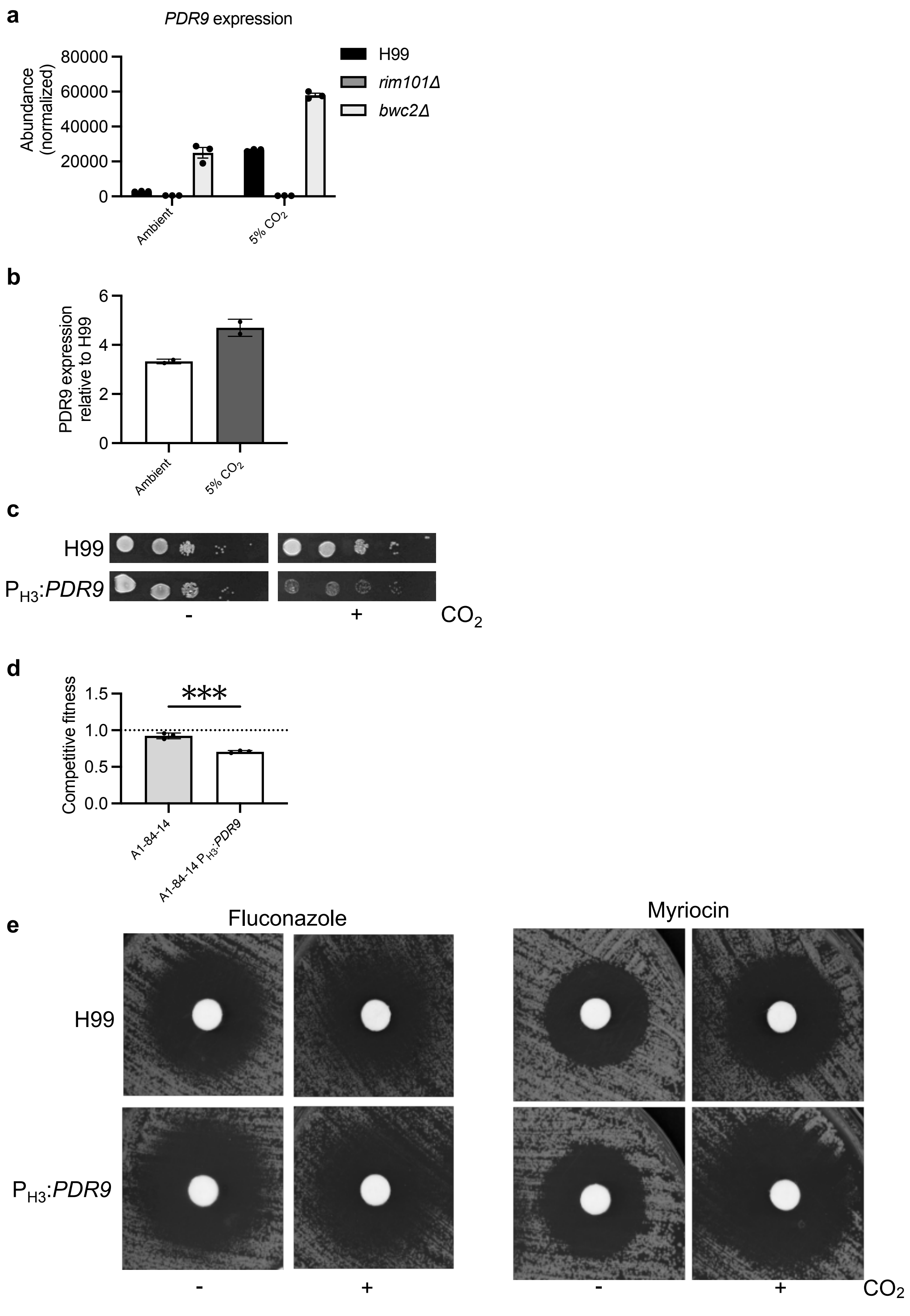
a
PDR9 expression
H99
rim101Δ
bwc2Δ
Abundance (normalized)
80000
60000
40000
20000
0
Ambient
5% CO2
b
PDR9 expression relative to H99
6
4
2
0
Ambient
5% CO2
c
H99
PH3:PDR9
-
+
CO2
d
Competitive fitness
1.5
1.0
0.5
0.0
***
A1-84-14
A1-84-14 PH3:PDR9
e
Fluconazole
Myriocin
H99
PH3:PDR9
-
+
-
+
CO2

Supplementary Fig. 3

Elevated absolute expression of *PDR9* confers CO_2 sensitivity

a) H99, *rim101*Δ and *bwc2*Δ strains were cultured in RPMI 1640 medium with 165 mM MOPS, pH 7 for 24 hours at 37°C in ambient air or 5% CO_2. Total RNA was isolated from harvested cells. 100 ng of RNA was hybridized to a custom Nanostring probe set and quantified on a Nanostring Sprint nCounter. Normalized Nanostring counts from total RNA for *PDR9* displayed for three biological replicates. b) Overnight cultures of H99 and P_{H3}:*PDR9* were washed and diluted to $7.5x10^5$ cells/ml and cultured in RPMI 1640 medium with 165 mM MOPS, pH 7 for 4 hours at 37°C in ambient air or 5% CO_2. *PDR9* gene expression was measured from total RNA and normalized to actin expression. P_{H3}:*PDR9* normalized expression is displayed relative to H99 for each condition. Measurements were performed in technical duplicates for biological duplicates. c) Ten-fold serial dilutions from overnight cultures of indicated strains were spotted on solid RPMI medium with 165 mM MOPS, pH 7 at 30°C in ambient air or at 5% CO_2 for 48 hours. Images are representative of three biological replicates. d) Overnight cultures of mNeon-Green labeled H99 and A1-84-14 or A1-84-14 P_{H3}:*PDR9* were combined in a 1:1 ratio and incubated at 30°C for 24 hours in ambient air or with 5% CO_2. Cell populations were characterized by flow cytometry and the percentage of mNeonGreen negative cells in 5% CO_2 normalized to those in ambient conditions to determine a competitive fitness score for each mutant strain. Bars represent the average and SEM of three biological replicates. A two-sided, unpaired t test was performed using GraphPad Prism. ***, $P < 0.001$. P=0.0009. e) Cells from overnight cultures of H99 and P_{H3}:*PDR9* were spread on RPMI 1640 medium with 165 mM MOPS, pH 7. Sterile disks were placed on plates and 40 µg fluconazole or 8 µg myriocin was added to each disk. Cells were incubated at 30°C for 48 hours in ambient air or 5% CO_2. Images are representative of two biological replicates. Source data are provided as a Source Data file.

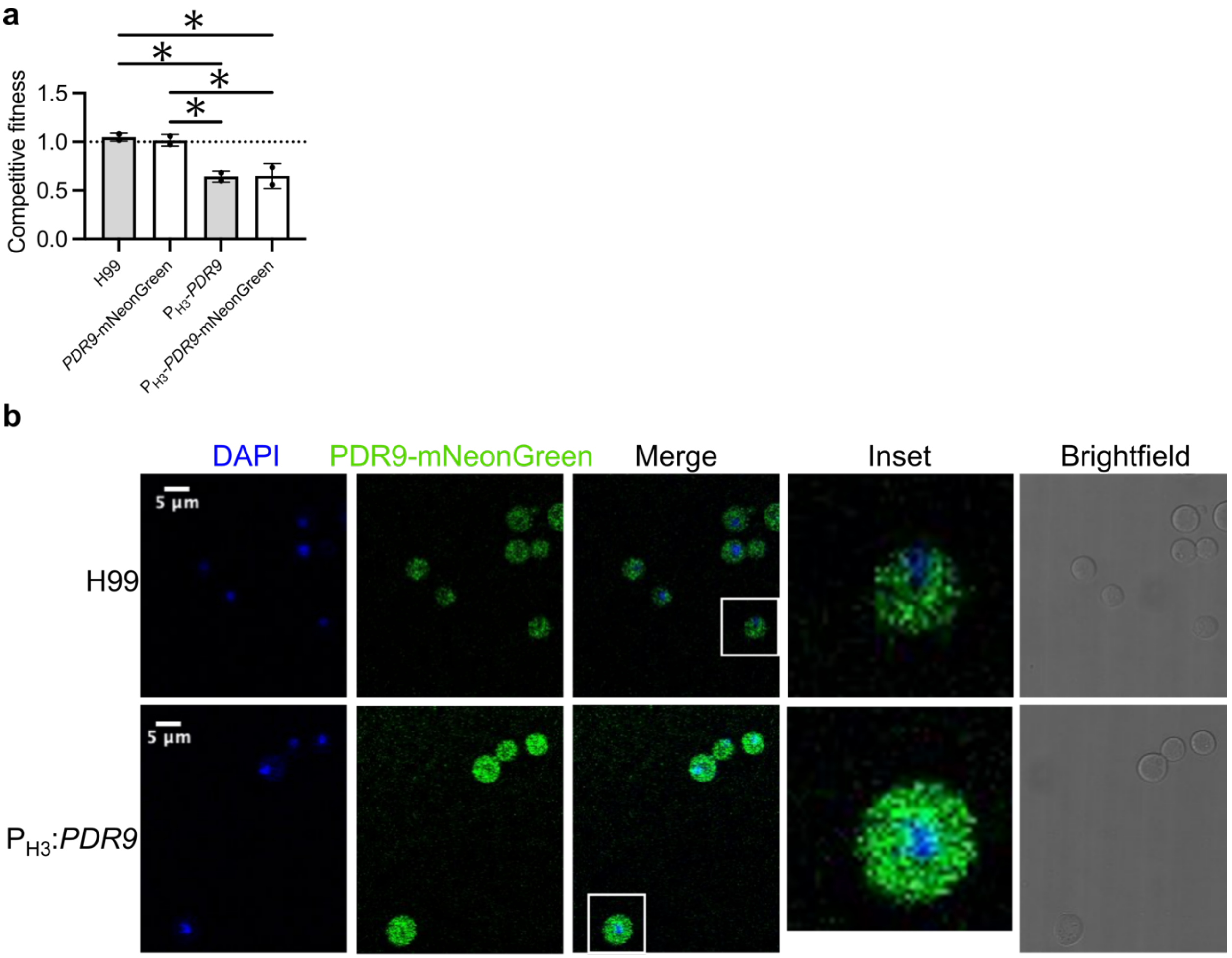

Supplementary Fig. 4

Putative PDR9 localization in the late Golgi/endosome

a) Overnight cultures of mNeon-Green labeled H99 and indicated strains were combined in a 1:1 ratio and incubated at 30°C for 24 hours in ambient air or with 5% CO_2. Cell populations were characterized by flow cytometry and the percentage of mNeonGreen negative cells in 5% CO_2 normalized to those in ambient conditions to determine a competitive fitness score for each strain as indicated (fluorescence intensity of PDR9-mNeonGreen is not high enough to obscure a difference between mNeon-Green labeled H99 and PDR9-mNeonGreen expressing strains). Bars represent the average and SEM of biological duplicates. An ordinary one-way ANOVA with Tukey's multiple comparisons test was performed using GraphPad Prism. *, $P < 0.05$. $P_{H99vs.\ PH3-PDR9-mNeonGreen}=0.0246$; $P_{H99vs.PH3-PDR9}=0.0230$; $P_{PDR9-mNeonGreenvs.PH3-PDR9-mNeonGreen}=0.0327$; $P_{PDR9-mNeonGreenvs.PH3-PDR9}=0.0305$. b) Mid-log phase H99 or P_{H3}:*PDR9* cells expressing mNeonGreen tagged PDR9 were stained with NucBlue (Hoechst 33342), and images were captured on a Leica confocal microscope. At least 50 cells per strain were examined and representative localization is displayed. Source data are provided as a Source Data file.

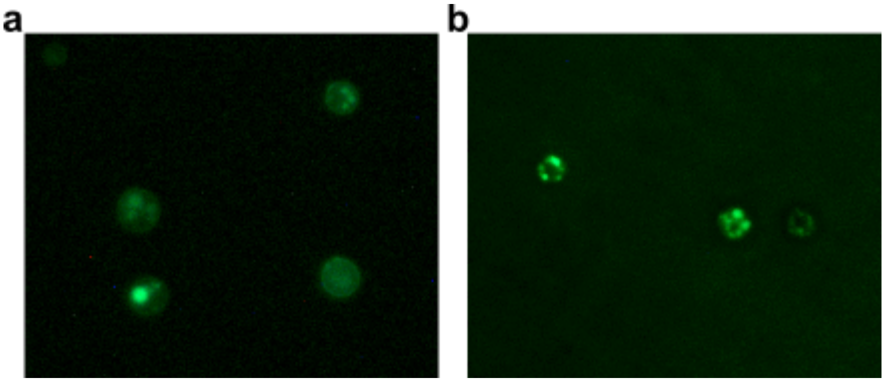

Supplementary Fig. 5

NBD-labeled lipids are internalized by cells

Cells from overnight cultures of H99 were cultured in RPMI 1640 medium with 165mM MOPS, pH 7 at 30°C in ambient air for 18 hours to mid-log phase. Cells were washed and labeled with NBD-phosphatidylserine (a) or NBD-phosphatidylethanolamine (b) for 30 minutes before washing and assessment of localization by microscopy. At least 50 cells per condition were examined and representative localization is displayed.

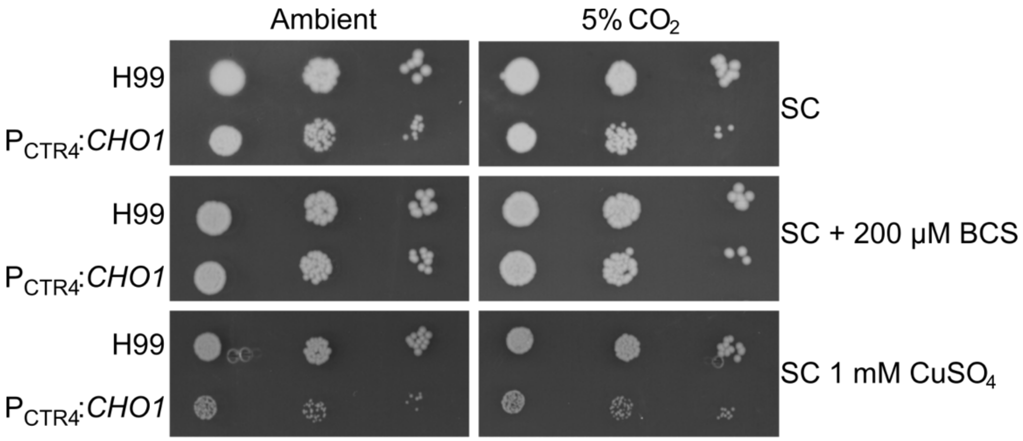

Supplementary Fig. 6

Elevated exposed PS does not confer CO_2 sensitivity
Ten-fold serial dilutions from overnight cultures of indicated strains were spotted on SC alone or with additions as indicated at 37°C in ambient air or at 5% CO_2 for 96 hours. Images are representative of biological duplicates.

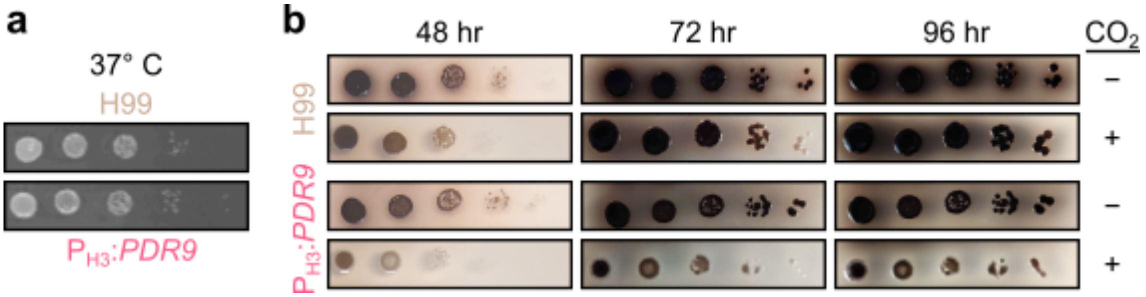

Supplementary Fig. 7

Constitutive expression of PDR9 does not induce defects in survival at host temperature or melanin production

a) Ten-fold serial dilutions from overnight cultures of indicated strains were spotted on solid RPMI medium with 165 mM MOPS, pH 7 at 37°C in ambient air or at 5% CO_2 for 48 hours. Images are representative of biological duplicates. b) Washed cells from overnight cultures were spotted on melanin-inducing agar medium and incubated at 30°C in ambient air or 5% CO_2 before imaging at indicated time points. Images are representative of biological duplicates.

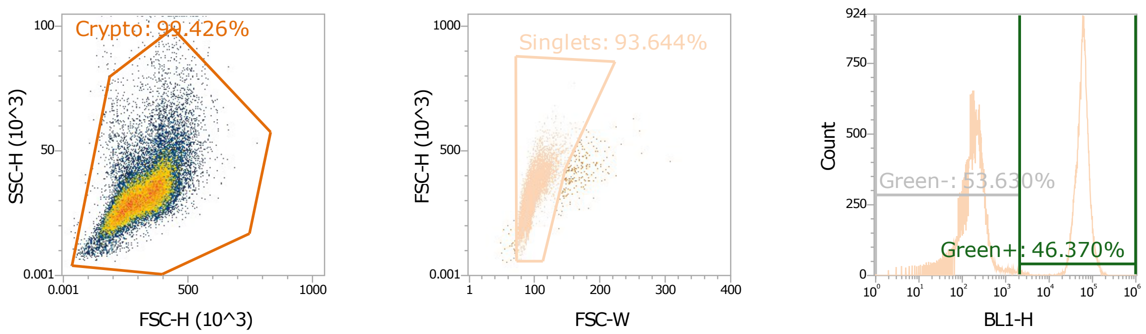

Supplementary Fig. 8

Flow cytometry gating strategy for competition assay

From SSC-H (Y) vs. FSC-H (X), intact cells were gated. This population was set to FSC-H (Y) vs. FSC-W (X) and single cells were gated. This population was plotted on a histogram plot of the BL1 laser (530/30) and the populations to the left of the valley (Green-) were quantified in a histogram gate. mNeonGreen+ cells were consistently completely separated from unlabeled cells by a 0.5 log valley. The percentages of mNeonGreen- cells varied by CO2 phenotype of the strains tested and was used to compare CO2+ survival to ambient air survival, resulting in bar graphs plotted in the figures.
