# Supplementary material for: Cryptococcus neoformans adapts to the host environment through TOR-mediated remodeling of phospholipid asymmetry
Source: Nat Commun. 2023 Oct 18;14:6587. doi: 10.1038/s41467-023-42318-y (PMC10584969; doi:10.1038/s41467-023-42318-y)
Supplement: Supplementary file 3 — Description of Additional Supplementary Files [file 41467_2023_42318_MOESM3_ESM.pdf]

Description of Additional Supplementary Files

Supplementary Data 1: Protein kinase screen results. Statistical significance was determined by CHI square test in Microsoft Excel. If mutant strains were deficient in ambient conditions (below 10% of the population at 24 hours) a two-sided Student's t-test was used to compare the population in CO2 to ambient.

Supplementary Data 2: Transcription factor screen results. Statistical significance was determined by CHI square test in Microsoft Excel. If mutant strains were deficient in ambient conditions (below 10% of the population at 24 hours) a two-sided Student's t-test was used to compare the population in CO2 to ambient.

Supplementary Data 3: H99 CO2-Amb RNA-Seq time course. Differential expression fold change, Wald test p values, and Benjamini-Hochberg adjustment for multiple comparisons are reported.

Supplementary Data 4: GO-term analysis. FDR < 0.05, Benjamini-Hochberg.

Supplementary Data 5: RIM101 CO2 vs H99 CO2 RNA-Seq. Differential expression fold change, Wald test p values, and Benjamini-Hochberg adjustment for multiple comparisons are reported.

Supplementary Data 6: Nanostring probe set – raw and processed results

Supplementary Data 7: Strains used in this study

Supplementary Data 8: Primers used in this study

Supplementary Data 9: Plasmid sequence and annotations for NAT-P_{H3} construct
