# Supplementary material for: Cryptococcus neoformans adapts to the host environment through TOR-mediated remodeling of phospholipid asymmetry
Source: Nat Commun. 2023 Oct 18;14:6587. doi: 10.1038/s41467-023-42318-y (PMC10584969; doi:10.1038/s41467-023-42318-y)
Supplement: Supplementary file 13 — Reporting Summary [file 41467_2023_42318_MOESM13_ESM.pdf]

nature portfolio

Corresponding author(s): Damian Krysan

Last updated by author(s): 9/14/2023

Reporting Summary

Nature Portfolio wishes to improve the reproducibility of the work that we publish. This form provides structure for consistency and transparency in reporting. For further information on Nature Portfolio policies, see our Editorial Policies and the Editorial Policy Checklist.

Statistics

For all statistical analyses, confirm that the following items are present in the figure legend, table legend, main text, or Methods section.

n/a	Confirmed	
☐	☒	The exact sample size (*n*) for each experimental group/condition, given as a discrete number and unit of measurement
☐	☒	A statement on whether measurements were taken from distinct samples or whether the same sample was measured repeatedly
☐	☒	The statistical test(s) used AND whether they are one- or two-sided<br>*Only common tests should be described solely by name; describe more complex techniques in the Methods section.*
☒	☐	A description of all covariates tested
☐	☒	A description of any assumptions or corrections, such as tests of normality and adjustment for multiple comparisons
☐	☒	A full description of the statistical parameters including central tendency (e.g. means) or other basic estimates (e.g. regression coefficient) AND variation (e.g. standard deviation) or associated estimates of uncertainty (e.g. confidence intervals)
☐	☒	For null hypothesis testing, the test statistic (e.g. *F*, *t*, *r*) with confidence intervals, effect sizes, degrees of freedom and *P* value noted<br>*Give P values as exact values whenever suitable.*
☒	☐	For Bayesian analysis, information on the choice of priors and Markov chain Monte Carlo settings
☒	☐	For hierarchical and complex designs, identification of the appropriate level for tests and full reporting of outcomes
☐	☒	Estimates of effect sizes (e.g. Cohen's *d*, Pearson's *r*), indicating how they were calculated

Our web collection on statistics for biologists contains articles on many of the points above.

Software and code

Policy information about availability of computer code

Data collection	Imaging data was collected on LAS X software version 3.5.7.23225.<br>Flow cytometry was collected in Attune Cytometric Software 5.3.2415.0.<br>RNASeq was performed by Azenta Life Sciences. As reported by the company, image analysis and base calling were conducted by the HiSeq Control Software (HCS). Raw sequence data (.bcl files) generated from Illumina HiSeq was converted into fastq files and de-multiplexed using Illumina bcl2fastq 2.20 software. Paired-end Illumina sequence read files were evaluated for quality and the absence of adaptor sequence using FastQC (https://www.bioinformatics.babraham.ac.uk/projects/fastqc/). Read files were mapped to C. neoformans reference genome H99 v48 (FungiDB) and gene transcript expression was quantified using HISAT2 (http://daehwankimlab.github.io/hisat2/) and Stringtie (https://ccb.jhu.edu/software/stringtie/). Differential expression fold change, Wald test p values, and Benjamini-Hochberg adjustment for multiple comparisons were determined using DESeq2(https://bioconductor.org/packages/release/bioc/html/DESeq2.html).<br>Chemiluminescent blots were imaged on a myECL imager, version 2.2.0.1250.<br>qRT-PCR data was collected on a Bio-Rad CFX Connect Real Time PCR Detection System with CFX Maestro software 2.3, v.5.3.022.1030.
Data analysis	Mean fluorescence intensity and capsule size were determined in ImageJ Fiji software version 1.53k.<br>Nanostring rcc files were extracted in nSolver software version 4.0 and analyzed in Microsoft Excel version 16.76.<br>Normalized counts were used to generate a heat map in Morpheus (https://software.broadinstitute.org/morpheus).<br>Statistics and graphing were performed in Microsoft Excel version 16.76 or GraphPad Prism v9.

For manuscripts utilizing custom algorithms or software that are central to the research but not yet described in published literature, software must be made available to editors and reviewers. We strongly encourage code deposition in a community repository (e.g. GitHub). See the Nature Portfolio guidelines for submitting code & software for further information.

Data

Policy information about availability of data

All manuscripts must include a data availability statement. This statement should provide the following information, where applicable:

- Accession codes, unique identifiers, or web links for publicly available datasets
- A description of any restrictions on data availability
- For clinical datasets or third party data, please ensure that the statement adheres to our policy

The RNA-Seq data discussed in this publication have been deposited in NCBI's Gene Expression Omnibus (58) and are accessible through GEO Series accession number GSE 241788 (https://www.ncbi.nlm.nih.gov/geo/query/acc.cgi?acc=GSE 241788). H99 reference genome v48 is available via FungiDB (https://fungidb.org/common/downloads/release-48/CneoformansH99/). The data supporting the findings of this work are provided within the article and supplementary files. Source data are provided with this paper. Materials generated in this study will be made available upon request, as there is no public repository for Cryptococcus neoformans strains.

Research involving human participants, their data, or biological material

Policy information about studies with human participants or human data. See also policy information about sex, gender (identity/presentation), and sexual orientation and race, ethnicity and racism.

Reporting on sex and gender	N/A
Reporting on race, ethnicity, or other socially relevant groupings	N/A
Population characteristics	N/A
Recruitment	N/A
Ethics oversight	N/A

Note that full information on the approval of the study protocol must also be provided in the manuscript.

Field-specific reporting

Please select the one below that is the best fit for your research. If you are not sure, read the appropriate sections before making your selection.

☒ Life sciences ☐ Behavioural & social sciences ☐ Ecological, evolutionary & environmental sciences

For a reference copy of the document with all sections, see nature.com/documents/nr-reporting-summary-flat.pdf

Life sciences study design

All studies must disclose on these points even when the disclosure is negative.

Sample size	In vitro experiments were performed in biological triplicate, as indicated in figure legends and methods. Studies involving flow cytometry collected 100,000 cells for each individual sample. Mouse studies used five mice in the co-infection experiment and ten mice per group in the survival study. No sample size calculation was performed; sample sizes were chosen based on previously published protocols and consensus in the field.
Data exclusions	No data were excluded from the analyses.
Replication	Experimental assays were performed in at least three independent replicates. qRT-PCR included two technical replicates for each of the three biological replicates. Disk diffusion assays were completed in duplicate or triplicate and one representative image is displayed. All attempts at replication were successful.
Randomization	Randomization was not relevant to in vitro studies as we were performing a uniform set of techniques directly comparing a labeled wild-type to mutant strain in competition assays or comparing the phenotype of a wild-type and mutant strain in the same assay under the same conditions. For animal studies, mice were received from a major supplier, and were chosen randomly for infection with C. neoformans wild-type or mutant strains. All animals were housed under the same conditions.
Blinding	Experiments were not performed blinded. Experiments generated quantitative measurements that did not require qualitative analyses which may introduce bias. All samples were treated uniformly and without remeasurement following data collection or analysis. All experiments were performed without discrimination.

Reporting for specific materials, systems and methods

We require information from authors about some types of materials, experimental systems and methods used in many studies. Here, indicate whether each material, system or method listed is relevant to your study. If you are not sure if a list item applies to your research, read the appropriate section before selecting a response.

Materials & experimental systems

n/a	Involved in the study
☐	☒ Antibodies
☐	☒ Eukaryotic cell lines
☒	☐ Palaeontology and archaeology
☐	☒ Animals and other organisms
☒	☐ Clinical data
☒	☐ Dual use research of concern
☒	☐ Plants

Methods

n/a	Involved in the study
☒	☐ ChIP-seq
☐	☒ Flow cytometry
☒	☐ MRI-based neuroimaging

Antibodies

Antibodies used	anti-p-p44 (phospho-p44/42 MAPK, catalog no. 4370; Cell Signaling)<br>mouse anti-GFP clones 7.1 and 13.1 (catalog no. 118144600001; Sigma)<br>goat anti-mouse horseradish peroxidase (HRP) (catalog no. STAR207P; Bio-Rad)<br>goat anti-rabbit horseradish peroxidase (HRP) (catalog no. STAR208P; Bio-Rad)<br>monoclonal antibody 18b7 (gift from A. Casadevall, 57. Mukherjee, J., Casadevall, A. & Scharff, M. D. Molecular characterization of the humoral responses to Cryptococcus neoformans infection and glucuronoxylomannan-tetanus toxoid conjugate immunization. J. Exp. Med. 177, 1105–1116 (1993).)
Validation	anti-p-p44 from the manufacturer: Monoclonal antibody is produced by immunizing animals with a synthetic phosphopeptide corresponding to residues surrounding Thr202/Tyr204 of human p44 MAP kinase. Phospho-p44/42 MAPK (Erk1/2) (Thr202/Tyr204) (D13.14.4E) XP® Rabbit mAb detects endogenous levels of p44 and p42 MAP Kinase (Erk1 and Erk2) when dually phosphorylated at Thr202 and Tyr204 of Erk1 (Thr185 and Tyr187 of Erk2), and singly phosphorylated at Thr202. This antibody does not cross-react with the corresponding phosphorylated residues of either JNK/SAPK or p38 MAP kinases.<br>mouse anti-GFP from the manufacturer: Monoclonal antibody for detection of both wild-type and mutant forms of GFP or GFP fusions. Anti-GFP is tested for functionality and purity relative to a reference standard to confirm the quality of each new reagent preparation. Purity: Both Anti-GFP mouse monoclonal antibodies (Clones 7.1 and 13.1) are >95% pure as determined by SDS-PAGE and ion-exchange HPLC analyses.

Eukaryotic cell lines

Policy information about cell lines and Sex and Gender in Research

Cell line source(s)	J774A.1 mouse Balb/C monocyte macrophage from a female mouse, purchased from ATCC (CB_91051511) in 2021.
Authentication	J774A.1 cell line was purchased from ATCC (CB_91051511). We did not authenticate it upon receipt.
Mycoplasma contamination	Cell lines were not tested for mycoplasma contamination.
Commonly misidentified lines<br>(See ICLAC register)	No commonly misidentified cell lines were used in this study.

Animals and other research organisms

Policy information about studies involving animals; ARRIVE guidelines recommended for reporting animal research, and Sex and Gender in Research

Laboratory animals	Six- to eight-week-old female CD1 mice.
Wild animals	This study didn't involve wild animals.
Reporting on sex	According to established protocols for C. neoformans infections, the sex of model animals does not influence the outcome of experiments, therefore female mice were chosen for increased ease of handling (Lim, T. S., Murphy, J. W. & Cauley, L. K. Host-etiological agent interactions in intranasally and intraperitoneally induced Cryptococcosis in mice. Infect. Immun. 29, 633–641 (1980); Lortholary, O., Improvisi, L., Fitting, C., Cavaillon, J.-M. & Dromer, F. Influence of gender and age on course of infection and cytokine responses in mice with disseminated Cryptococcus neoformans infection. Clin. Microbiol. Infect. 8, 31–37 (2002).
Field-collected samples	This study didn't involve samples collected from the field.

Ethics oversight

This study was performed according to the guidelines of NIH and the University of Georgia Institutional Animal Care and Use Committee (IACUC). The animal models and procedures used have been approved by the IACUC (AUP protocol number: A2020 06-015).

Note that full information on the approval of the study protocol must also be provided in the manuscript.

Flow Cytometry

Plots

Confirm that:

- [ ] The axis labels state the marker and fluorochrome used (e.g. CD4-FITC).
- [ ] The axis scales are clearly visible. Include numbers along axes only for bottom left plot of group (a 'group' is an analysis of identical markers).
- [ ] All plots are contour plots with outliers or pseudocolor plots.
- [ ] A numerical value for number of cells or percentage (with statistics) is provided.

Methodology

Sample preparation	*No flow plots are presented in this article. Quantitative data was acquired through flow cytometry to characterize survival of various strains in ambient or CO2 conditions.<br><br>Overnight cultures of yeast were combined in a 1:1 ratio of fluorescent H99 with unlabeled experimental strain and grown for 24 hours in a 96-well plate at 30°C in ambient air or with 5% CO2 before reading on the flow cytometer.
Instrument	Thermo Fisher Scientific Attune Flow Cytometer
Software	Attune NxT flow cytometry software
Cell population abundance	100,000 cells were collected for each sample in the "singlets" gate which functioned to exclude debris, budding cells or clumps of cells. The singlets gate was consistently 70%+ of the total events in each sample.
Gating strategy	From SSC-H (Y) vs. FSC-H (X), intact cells were gated. This population was set to FSC-H (Y) vs. FSC-W (X) and single cells were gated. This population was plotted on a histogram plot of the BL1 laser (530/30) and the populations to the left of the valley (Green-) were quantified in a histogram gate. mNeonGreen+ cells were consistently completely separated from unlabeled cells by a 0.5 log valley. The percentages of mNeonGreen- cells varied by CO2 phenotype of the strains tested and was used to compare CO2+ survival to ambient air survival, resulting in bar graphs plotted in the figures.

- [x] Tick this box to confirm that a figure exemplifying the gating strategy is provided in the Supplementary Information.
